# The effect of adapting Hospital at Home to facilitate implementation and sustainment on program drift or voltage drop

**DOI:** 10.1186/s12913-019-4063-8

**Published:** 2019-04-29

**Authors:** Albert L. Siu, Robert M. Zimbroff, Alex D. Federman, Linda V. DeCherrie, Melissa Garrido, Barbara Morano, Sara Lubetsky, Elisse Catalan, Bruce Leff

**Affiliations:** 1 Brookdale Department of Geriatrics and Palliative Medicine, Icahn School of Medicine at Mount Sinai, 1 Gustave L. Levy Place, New York, NY 10029 USA; 2 Icahn School of Medicine at Mount Sinai, New York, USA; 3 Division of General Internal Medicine, Department of Medicine, Icahn School of Medicine at Mount Sinai, New York, NY USA; 4 Division of Geriatrics, Department of Medicine, Johns Hopkins University School of Medicine, Baltimore, Maryland USA; 5 James J. Peters VA Medical Center, New York, NY USA; 6 Department of Health Law, Policy & Management, Boston University School of Public Health, Boston, Massachusetts USA

**Keywords:** Implementation, Adaptation, Health outcome assessment, Quality of care

## Abstract

**Background:**

Translating evidence-based interventions from study conditions to actual practice necessarily requires adaptation. We implemented an evidence-based Hospital at Home (HaH) intervention and evaluated whether adaptations could avoid diminished benefit from “voltage drop” (decreased benefit when interventions are applied under more heterogeneous conditions than existing in studies) or “program drift.” (decreased benefit arising from deviations from study protocols).

**Methods:**

Patients were enrolled in HaH over a 6-month pilot period followed by nine quarters of implementation activity. The program retained core components of the original evidence-based HaH model, but adaptations were made at inception and throughout the implementation. These adaptations were coded as to who made them, what was modified, for whom the adaptations were made, and the nature of the adaptations. We collected information on length of stay (LOS), 30-day readmissions and emergency department (ED) visits, escalations to the hospital, and patient ratings of care. Outcomes were assessed by quarter of admission. Selected outcomes were tracked and fed back to the program leadership. We used logistic or linear regression with an independent variable included for the numerical quarter of enrollment after the initial 6-month pilot phase. Models controlled for season and for patient characteristics.

**Results:**

Adaptations were made throughout the implementation period. The nature of adaptations was most commonly to add or to substitute new program elements. HaH services substituting for a hospital stay were received by 295 patients (a mean of 33, range 11–44, per quarter). A small effect of quarter from program inception was seen for escalations (OR 1.09, 95% CI 1.01 to 1.18, *p* = 0.03), but no effect was observed for LOS (− 0.007 days/quarter; SE 0.02, *p* = 0.75), 30 day ED visit (OR 0.93, 95% CI 0.86 to 1.01, *p* = 0.09), 30-day readmission (OR 1.00, 95% CI 0.93 to 1.08, *p* = 0.99), or patient rating of overall hospital care (OR for highest overall rating 0.99, 95% CI 0.93 to 1.05, *p* = 0.66).

**Conclusions:**

We made adaptations to HaH at inception and over the course of implementation. Our findings indicate that adaptations to evidence-based programs may avoid diminished benefits due to potential ‘program drift’ or ‘voltage drop.’

**Trial registration:**

Not applicable. This study is not a clinical trial by the International Committee of Medical Journal Editors (ICMJE) definition because it is an observational study “in which the assignment of the medical intervention is not at the discretion of the investigator.”

**Electronic supplementary material:**

The online version of this article (10.1186/s12913-019-4063-8) contains supplementary material, which is available to authorized users.

## Background

Translating complex multi-component evidence-based interventions from study conditions and protocols to actual practice necessarily requires minor to major adaptation of intervention content, format, personnel roles and processes. Adaptations may be desired or necessary to customize to local circumstances or to account for new technology or temporal changes in disease epidemiology or clinical practice [1, 2]. Traditionally, it has been thought that variance from an evidence-based protocol is accompanied with at least some diminution of effectiveness. This effect has been termed “program drift” (i.e., decreased benefit arising from deviations from study protocols) and “voltage drop” (i.e., loss of benefit when interventions are applied in more heterogeneous patient populations and settings as they move from efficacy to effectiveness and into actual practice) [3].

More recently, the inevitability of diminished benefits due to program drift and voltage drop has been questioned. While fidelity to a study population, setting, and protocol may be critical for a biological or a clinical intervention to maintain the studied benefits, this may not be the case for health system interventions or more complex multi-component interventions. Adaptations may be made as the intervention is applied in more heterogenous patient populations or practice settings, thereby potentiating voltage drop. Other adaptations may be made to the intervention protocol to improve fit with the practice setting and the environment, thereby potentiating program drift. Other adaptations may address technological or medical advances that were not available or formally manualized at the time of the evidence-based study. In theory, these adaptations may have neutral effects. Some adaptations may enhance the effectiveness of the original intervention^3,^ thereby neutralizing any diminished effectiveness from program drift or voltage drop or perhaps even increasing the net benefit beyond that observed in evidence-based studies. Other adaptations may inadvertently diminish benefits. Indeed, some have called for the systematic study of adaptations to evidence-based interventions and a formalized structure for updating the evidence base to account for adaptation [4].

To improve our understanding of the effect of adaptations, we took advantage of an implementation of an evidence-based hospital admission avoidance Hospital at Home (HaH) intervention [5]. This implementation necessitated extensive adaptation of studied procedures to local setting and culture and evolving medical practice. In 2014, The Mount Sinai Health System, a seven-hospital system in New York City, began implementing HaH with 30-day post-acute care follow-up of patients. For select patients with specific diagnoses (e.g., pneumonia) who would otherwise be admitted, HaH services (e.g., intravenous antibiotics, fluids, oxygen, etc.) and daily clinician visits are provided at home along with durable medical equipment, phlebotomy, and home x-ray as needed. We examined whether HaH associations with outcomes changed over time as adaptations were put into place.

Funded as an innovation award from the Center for Medicare and Medicaid Innovation, this implementation of HaH was instigated with the goal of eventual sustainment in the local health system and scaling and dissemination nationally. The phased plan for implementation within the local health system was consistent with the concepts outlined in the Dynamic Sustainability Framework (DSF) [3]. The DSF posits that interventions should be implemented with consideration of fit with practice settings and the external ecological system of competitors, regulation, market forces, and population needs. The framework also puts forth that these elements are not static and will change with time. Our phased implementation anticipated a piloting phase followed by initial implementation in one hospital emergency department with phased expansion over several quarters to other health system settings. We anticipated the need to adapt HaH to each hospital’s unique culture, medical staff, electronic record system, community, and labor practices. Similarly, we anticipated that HaH might need to be adapted to changing policy and regulations, different payers, and a healthcare market in dynamic flux due, in part, to the implementation of the Affordable Care Act.

## Methods

### Patients and settings

Patients were enrolled in HaH starting in November 2014 for 33 months through August 2017. An initial 6-month run-in pilot period was followed by nine quarters of implementation activity.

Details of HaH patient engagement procedures have been reported elsewhere [6]. Briefly, potentially eligible patients were identified in the emergency departments of Mount Sinai Health System hospitals, or by referral from physicians in outpatient clinical practices or a home-based primary care practice.

Patients were eligible for HaH if they were ≥ 18 years of age, required inpatient admission, and had fee-for-service Medicare or coverage from a single private insurer that contracted with Mount Sinai for HaH services. Patients were excluded if they were clinically unstable (e.g., had very low blood pressure), required cardiac monitoring or intensive care, lived in an unsafe home environment, or resided outside the specified catchment area.

### Hospital at Home, Core components, and adaptations and their coding

The HaH program retained many of the core components of original evidence-based studies of HaH (see Table 1). These included a) targeting to participants who needed to be hospitalized; b) delivering hospital-level services at home instead of the hospital; c) daily registered nurse visits to the home; d) clinician (physician or nurse practitioner) home visits; and e) 24/7availability to patients and family members.

**Table 1:** Core Programmatic Elements of HaH from Studies Treating Multiple Diagnoses

Trial	Core Programmatic Elements of HaH					Summary of Outcomes			
	Targeting patient requiring hospitalization	Delivering hospital-level services at home	Daily RN visits	Clinician (MD, NP) home visits	24/7 availability to patients and family members	Length of Stay	Mortality	Satisfaction	Total Acute Cost
Stressman et al. (1996) [7]	✔	✔	✔^a^	✔	✔	^a^Average utilization fell 9.4% in HH group; rose 2.3% in geriatric control group	no comparison	96% report physician and nursing care was “very good” or “good”	“Total savings estimated to be $5.62 million”
Wilson et al. (1999) [8]	✔	✔	✔	✔	✔	8 days vs. 14.5 days for controls (median, *p* = 0.026)	25% vs. 31% for controls at 3 months (RR 0.82, 95% CI 0.52 to 1.28)	Total satisfaction on 6-item questionnaire: 15 for HaH vs. 12 for controls (*p* = .001)	£2594 vs. £3659 for controls (*p* = .011)
Caplan et al. (1999) [9]	✔	✔	✔	✔	✔	10.1 days vs. 7.4 days for controls (*p* > .05)	No significant difference between groups at 28 days	Caregiver satisfaction significantly higher amongst HaH group vs. hospital controls (difference − 0.8 on a 4-point scale, *P* < 0.0001), with 55 and 27% response rate, respectively.	$1764 vs. $3775 for controls
Harris et al. (2005) [10]	✔	✔	✔	✔	✔	8.8 days vs. 5.7 days days for controls (*p* < 0.0001)	Not reported	HaH patients rating satisfaction as ‘very good’ or ‘excellent’ vs. those in the hospital group - 83.0% versus 72.3%, *p* = .05. Relatives of HaH rating satisfaction as ‘very good’ or ‘excellent’ vs. those of controls - 66.7% versus 41.3%, *p* = .004	NZ$6524 vs. NZ$3525 for controls^a^
Leff et al. (2005) [5]	✔	✔	✔	✔	✔	3.2 days vs. 4.9 days for controls (*p* = .004)	0% vs. 3% for controls (*p* = .05)	Satisfaction of patients (median, 7 vs. 6 domains; p < 0.001) and family members (median, 6 vs. 5 domains; *p* < 0.001) was greater in the intervention group and remained statistically significant when controlled for covariates	$5081 vs. $7480 for controls (p < 0.001)
Cryer et al. (2012) [11]	✔	✔	✔	✔	✔	3.3 days vs. 4.5 days for controls	0.93% vs. 3.4% for controls	HCAHPS overall patient satisfaction mean score for HaH group of 90.7 exceeded the hospital score of 83.9 for comparable patients.	“19% lower”
Summerfelt et al. (2015) [12]	✔	✔	✔	✔	✔	3.64 days vs. 4.31 days for controls (*p* = .088)	2% vs 2% for controls (*p* = 0.86)	HaH patients reported higher overall satisfaction score (4.40 vs 4–.01; *P* = .001)	N/A

^a^Not all patients had daily RN visits

Adaptations to the original HaH model were made at inception and throughout the 33-month implementation to enhance enrollment, to add new hospital sites, to improve workflow, to enhance patient care, to extend hours of intake, and to take advantage of new opportunities (availability of new technology or services such as community paramedicine). As described later in the list of adaptations made to the HaH program, HaH service lines evolved to include palliative care at home, observation unit at home, and rehabilitation at home (see full list in Table 2). We used an established coding system to categorize who made the adaptations, what was modified, for whom the adaptations were made, and the nature of the adaptations [1].

**Table 2:** Adaptations of HaH Model

Adaptations	Rationale	Quarter of Initiation
Addition of 30-day post-acute transition component to the HaH model	To improve transitions of care, reduce preventable readmissions, and establish follow up with primary care	0 (inception)
Expansion of original target diagnoses and reduce exclusions (e.g., HIV exclusion) to reflect current medical practice	To enroll patients with a broader set of diagnoses who could be safely treated at home, per clinical judgment	1
Implementation of Palliative Care Unit at Home	To provide acute services at home consistent with stated goals of care for patients with advanced illness who would otherwise have been excluded from HaH	1
Collaborated with community paramedicine program to consult with HaH physicians by video for patients needing urgent visits in the home	To better evaluate and address urgent clinical needs and avoid unnecessary visits to the emergency department	1 then suspended due to bankruptcy of partner and restarted in 6 with new partner
Contracting for infusion services	To increase staffing flexibility in being to provide infusion services	2
Dedicated nurses hired	To increase availability and consistency of nursing staff for the program	2
Implementation of Observation at Home	To treat patients with observation services at home with the expectation that some of these patients would require more extended HaH services	3
Implementation of Rehabilitation at Home	To treat patients who would otherwise require admission to a subacute rehabilitation facility in the home setting	3
Expansion to new sites for enrollment along with developing new intake procedures customized for each site		3, 6, and 9
Adaptation of intake procedure for patients identified to need HaH services late at night by holding the patients overnight in the emergency department and transferring home in the morning	To capture and enroll patients presenting to the ED overnight	4
Launch of telehealth visits to supplement home visits	To increase the frequency and efficiency of clinician contacts in the home	4
Internalized major portions of pharmacy and lab services	To speed availability of services to be provided to patients in the home	4
Implemented new version of electronic medical record	To update an earlier version of a HaH-specific electronic medical record to improve documentation and communication	6
Dedicated physical therapist hired	To increase availability and consistency of physical therapy services for the program	6
Role created for nurse care coordinator	To triage patient needs and coordinate staff involved in home visits	8
Piloted weekend admissions	To increase service hours	8

### Data collection and measures

Patient-level data were collected for the purposes of performance monitoring and final evaluation of program performance. Interviews were conducted at the bedside in the emergency department (ED) (see Additional file 1 for instrument guide), with follow-up interviews conducted at 2 and 4 weeks after admission by phone (see Additional files 2 and 3 for instrument guide). We collected information on the following patient outcomes: length of stay (LOS), 30-day readmissions and emergency department (ED) visits, escalations (i.e., needing to suspend a HaH episode in the acute rather than post-acute phase to transfer the patient to the hospital), and patient ratings of care measured with the Hospital Consumer Assessment of Healthcare Providers and Systems (HCAHPS) survey. HCAHPS was scored per guidelines from the Centers for Medicare and Medicaid Services [13], specifically, the proportion of individuals who gave a top-box rating for the measure (highest possible rating), adjusted for age, education, interview language (Spanish or English), and general health. Interviewers also documented patient demographics, performance in 5 activities of daily living (ADL), and general health (rated poor to excellent). Functional impairment was defined as needing some help or unable to perform one or more of 12 ADLs. Data on all outcomes, with the exception of patient ratings, which were less available to us in a timely fashion, were monitored monthly with feedback to program staff for review and continuous quality improvement.

### Analysis

Because the outcomes collected were most relevant to hospital stays, we report outcomes for patients admitted to the program where the intervention was used to substitute for a hospital stay. That is, we excluded patients receiving rehabilitation at home (*n* = 264). We also exclude patients who received observation services for a single overnight period (*n* = 41). If hospital services were extended overnight past a second midnight for patients initially admitted to observation services, we considered the case to have been converted into a regular HaH stay and include them in our analyses; this simulates what would have happened if a patient had been admitted to a regular hospital observation unit and required services past the second midnight. Patients receiving palliative care services at home were receiving HaH for what substitutes as a hospital stay and are included as well. After exclusions, our sample includes data on 295 patients.

We present outcomes by quarter of HaH patient admission. We used logistic or linear regression (in the case of LOS) with an independent variable included for the numerical quarter of enrollment after an initial 6-month pilot phase. We estimated the average marginal effect for number of quarters since the program began (marginal effect per each additional quarter) for LOS. Odds ratios were estimated for the other models. The models controlled for season of HaH admission, patient age, sex, race, ethnicity, education, insurance type, functional impairment, and self-rated health.

More than 10% of data were missing for race and ethnicity (*n* = 64), physical function (*n* = 115), and general health (*n* = 84). To maximize the number of patients in the models, we used multiple imputation, modeling the probability of missing data for each variable on age, sex, race and ethnicity, education, insurance type, pre-acute physical function, and general health. Analyses were performed with SAS version 9.3 (SAS Institute, Cary, NC).

Because the data in these analyses were used for internal program evaluation and reporting to Medicare, their collection was exempt from Mount Sinai Institutional Review Board (IRB) review and patient consent was not required. We requested and received approval from our IRB to conduct a retrospective analysis of these data.

## Results

Over the 33 months of implementation, a series of program adaptations were implemented (see Table 2), including a) adding a 30-day post-acute transition component to the HaH model (at inception); b) expanding target diagnoses and modifying exclusions from those originally studied (Quarter 1) due to changes in medical practice (e.g., removed HIV exclusion from decade-old protocol of original HaH studies); c) outside contracting for certain services (e.g., infusion) rather than providing the service directly with program staff (Quarter 2); d) implementing HaH variations due to change in medical practice (e.g., palliative care unit at home [Quarter 1], observation unit services at home [Quarter 3]) or opportunity (e.g., rehabilitation at home services [Quarter 3]); e) adapting intake procedures (e.g., holding patients overnight in the emergency room [Quarter 4]) for patients entering the program late at night when delivering supplies to the home to initiate a HaH episode was less available; f) launching telehealth visits (Quarter 4); and g) changing staffing and staff roles (Quarters 2, 6, 8). Other more minor adaptations were made throughout the implementation period.

All adaptations were initiated by the program team (see Table 3). Modifications were most commonly made in format (or how the intervention was delivered) and in personnel and roles. Modifications were made at the level of the cohort (e.g., for patients who would otherwise have been admitted to an observation unit in the hospital), patient population (e.g., for patients with new diagnoses or with palliative care needs that would not have been eligible for the original HaH intervention) or organization (e.g., staffing modifications that affected the entire program regardless of hospital site). The nature of adaptations was most commonly to add or to substitute new program elements. In three instances, these adaptations were sufficiently extensive for the cohort or population that it involved integrating the HaH approach to additional patient populations (palliative care patients) or to other clinical constructs (e.g., observation unit services or postacute rehabilitation).

**Table 3:** Coding of Adaptations of HaH

Wiltsey-Stirman’s Coding of Modifications and Adaptations of Evidence-based Interventions ^a^			
Adaptation	What was modified	For whom/what are modifications made	Nature of the modification
Addition of 30-day post-acute transition component to the HaH model	Content (30-day transition services)	Cohort (done for all excepting patients with one payer)	Adding element (appending new transition services to the end of a HaH episode)
Expansion of original target diagnoses and reduce exclusions (e.g., HIV exclusion) to reflect current medical practice	Content (new diagnostic categories targeted)	Population (expanded patient population) and Organization and Network (added throughout the program)	Adding element (adding diagnoses not previously in most HaH programs thereby expanding the pool of eligible patients)
Implementation of Palliative Care Unit at Home	Content (new service) and population (format for identifying patients with palliative care needs)	Population (new patient population that would not have previously qualified for HaH)	Adding an element, and integrating the intervention into another approach (adding new modules to HaH incorporating palliative care principles and approach to better meet needs of new population)
Collaborated with community paramedicine program to consult with HaH physicians by video for patients needing urgent visits in the home	Format (how urgent visits managed) and personnel (how community paramedicine staff were used)	Organization/Network (done throughout the program)	Substituting an element (community paramedicine visits substituted for urgent clinician home visits or transport to the emergency department in certain cases)
Contracting for infusion services	Personnel (format for inclusion of vendor for infusion services)	Organization/Network (done throughout the program)	Integrating another approach (contracted infusion nursing that did not do other aspects of nursing added to supplement existing staff for additional visits involving only infusion)
Dedicated nurses hired	Personnel (format for registered nurse staffing)	Organization/Network (done throughout the program)	Substituting an element (pool of nurses also involved in other duties substituted with nurses dedicated to HaH)
Implementation of Observation at Home	Content (new service) and setting (how patients otherwise admitted to hospital observation unit identified and managed)	Cohort (new group of patients with observation needs)	Tailoring, integrating the intervention into another approach, and departing from the intervention (tweaking of intake procedure to admit observation unit candidates, incorporating observation service procedures, and earlier discharge of observation patients after one day with the option of converting patients to longer stay HaH, if indicated)
Implementation of Rehabilitation at Home	Content (new service) and setting and population (format for identifying and caring for patients needing subacute care)	Cohort/Population (new group of patients from inpatient hospital units and slated to be referred to skilled nursing facilities for subacute care)	Adding an element and integrating the intervention into another approach (new intake procedure to admit subacute care candidates and incorporating subacute care practices into HaH)
Expansion to new sites for enrollment along with developing new intake procedures customized for each site	Setting and personnel (how new sites identified and managed patients and how personnel roles were modified accordingly)	Hospital/ Organization (expansion to different hospitals within the organization)	Adding an element and substituting elements (adding new hospitals and substituting different procedures and different type of personnel, as well as roles, depending on existing procedures and resources at the new site)
Adaptation of intake procedure for patients identified to need HaH services late at night by holding the patients overnight in the emergency department and transferring home in the morning	Format (how intake and care procedures modified for after hours), setting and personnel (different staffing for overnight services in the hospital)	Organization/Network (done throughout the program)	Adding an element, substituting elements, and loosening structure (substituting services in the hospital overnight for services HaH would otherwise provide at home)
Launch of telehealth visits to supplement home visits	Format (how video telehealth visits were conducted) and personnel (staffing to assist patient with telehealth at home and clinician staffing for video telehealth visits)	Organization/Network (done throughout the program)	Substituting elements and loosening structure (allowed substitutions of some clinician home visits with video telehealth visits)
Internalized major portions of pharmacy and lab services	Format (how pharmacy and lab requests managed)	Organization/Network (done throughout the program)	Substituting an element (supplemented vendor services with option to use internal resources)
Implemented new version of electronic health record (EHR)	Format (how care was communicated and documented for HaH)	Hospital/ Organization (done throughout the program but with hospital-specific processes depending on EHR used)	Substituting an element (new version of HaH EHR with improved functionalities replacing previous version)
Dedicated physical therapist hired	Personnel (format for providing physical therapy services)	Organization/Network (done throughout the program)	Substituting an element (substituted full time dedicated physical therapist for pool of physical therapists also serving patients in other programs)
Role created for nurse care coordinator	Format (how HaH care coordinated) and personnel (new role for existing staff)	Organization/Network (done throughout the program)	Tailoring (nurse care coordinator to manage active patient cases and coordinate staff and services)
Piloted weekend admissions	Format and personnel (separate procedures, as well as personnel roles, for weekend admissions)	Organization/Network (done throughout the program)	Adding an element and loosening structure (new processes instituted for weekend admissions due to reduced weekend staff on duty)

^a^Wiltsey-Stirman’s coding also includes coding of by whom modifications are made. All adaptations were made by the program team [1]

HaH services substituting for a hospital stay were received by 295 patients. The average LOS was 3.2 days. Of our sample, 12.2% had escalations, 8.6% had readmissions within 30 days, and 5.8% had ED visits within 30 days. The overall rating for hospital care was rated at the highest level by 68.8% of patients.

**Fig. 1:**
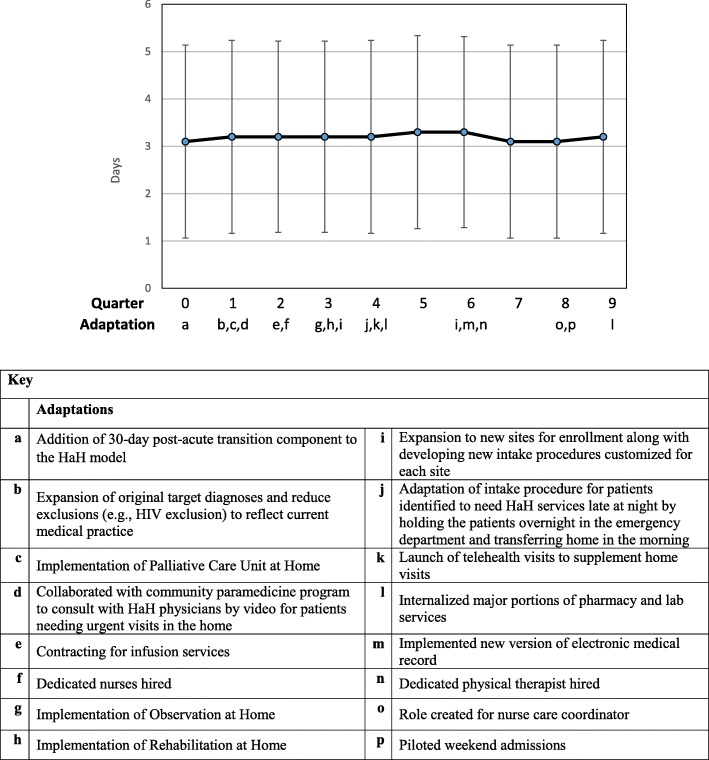
Mean Length of Stay (LOS) in days, Change over quarters SE 0.02, *p* = 0.75

**Fig. 2:**
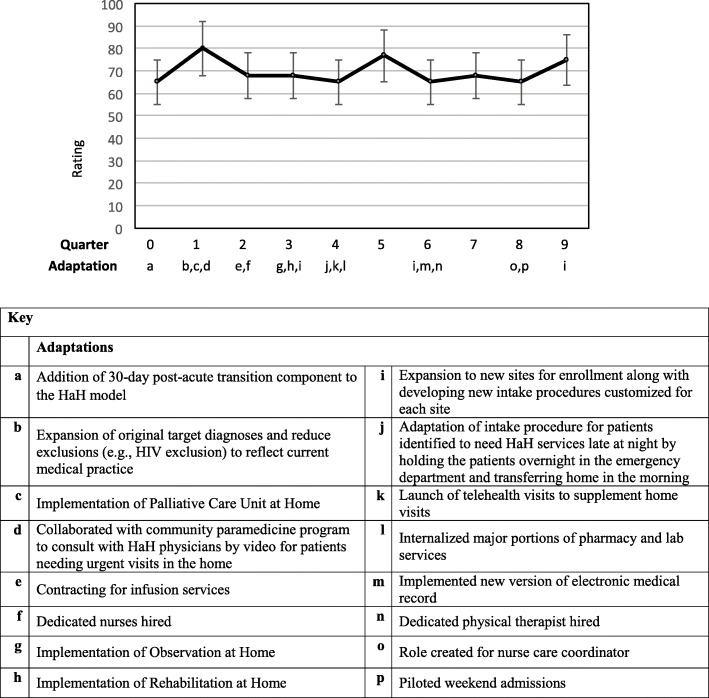
Hospital Rating (High) HCAHPS. Change over quarters OR 0.99, *p* = 0.66

**Fig. 3:**
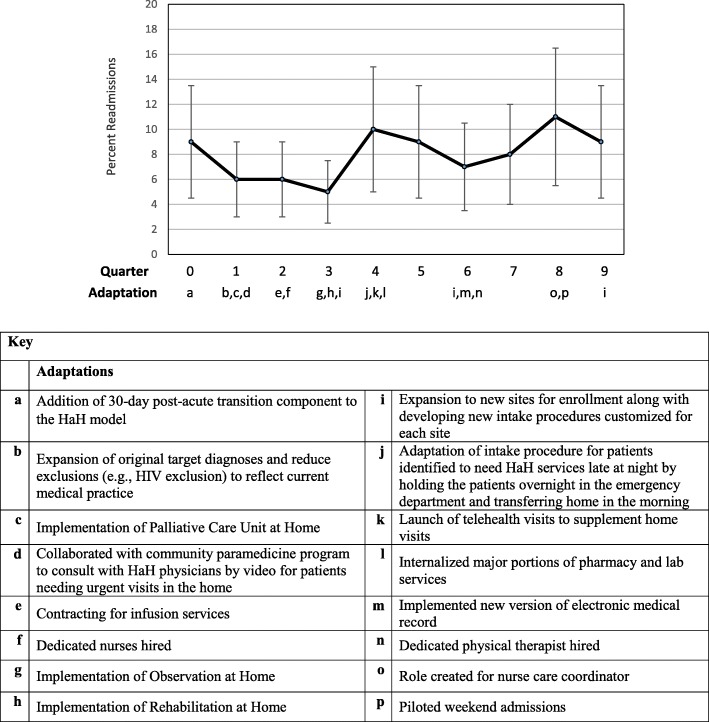
Percent Hospital Readmissions, Change over quarters, OR 1.00, *p* = 0.99

**Fig. 4:**
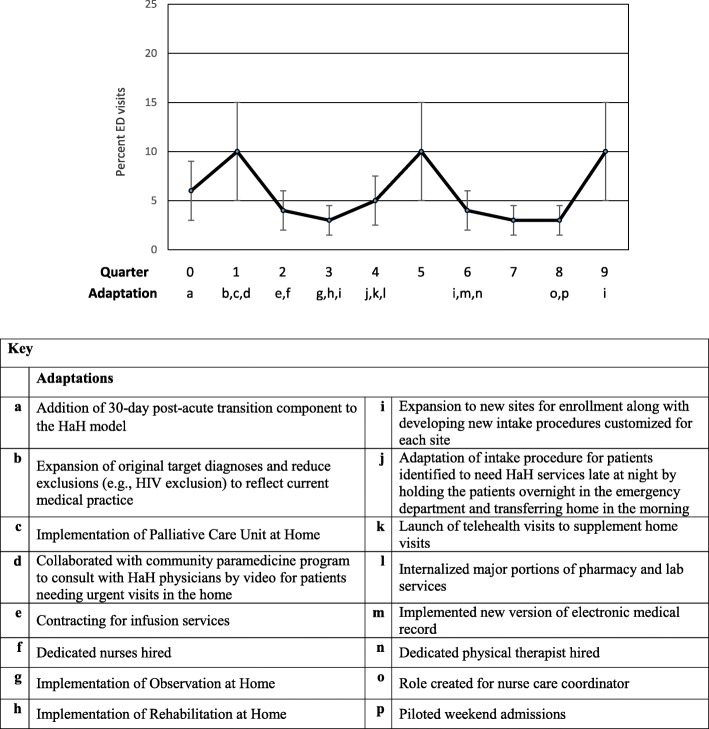
Percent ED Visits, Change over quarters, OR 0.93, *p* = 0.09

Graphs of these outcomes, adjusted for patient characteristics and season, over the nine quarters of implementation are provided in Figs. 1, 2, 3 and 4. A median of 33 patients (range 11–44) received services each quarter. The graphs show occasional spikes in events such as readmissions. Confidence intervals and standard errors were moderately large at any point in time; however they indicate that changes, if any, were small over time. The graphs indicate the quarter of initiation for the adaptations listed in Tables 2 and 3.

Regression models controlling for patient characteristics and season of admission are available in Additional file 4. Older age was associated with higher ED revisits and higher ratings of care. African American patients had shorter LOS and higher 30-day ED revisits and readmissions. Having Medicaid was associated with higher ED revisits and lower ratings of care. Poor self-rated health was associated with longer LOS, greater escalations, and higher ratings of care.

In the regressions, we did not observe a relationship between quarter from program inception and LOS (− 0.007 days/quarter; SE 0.02, *p* = 0.75). The odds ratios (OR) per quarter from program inception was mildly increased for escalations (OR 1.09, 95% CI 1.01 to 1.18, *p* = 0.03), but was not increased or decreased for 30 day ED visit (OR 0.93, 95% CI 0.86 to 1.01, *p* = 0.09), 30-day readmission (OR 1.00, 95% CI 0.93 to 1.08, *p* = 0.99), and the patient providing the highest rating for overall hospital care (OR 0.99, 95% CI 0.93 to 1.05, *p* = 0.66).

## Discussion

We made adaptations to an evidence-based model of HaH at program inception and throughout a 33-month implementation. Our experience reinforces concepts from the DSF. Many adaptations were made to improve HaH operations. Others were made to respond to patient needs and referring provider requests such as for palliative care patients who needed acute hospital-level care. Some adaptations were made to improve scalability such as extending intake hours into nights and weekends. Other adaptations were made because new opportunities became available to enhance the patient experience such as the initiation of video telehealth and community paramedicine visits. These adaptations would have been difficult to include at program inception or even to script into phased introductions as the availability of the new opportunities or need for adaptation was unknown at that time. In some cases, adaptations were necessary to adapt to differing practice contexts at different hospitals. Expansion to other hospitals usually required new intake procedures to adjust to local culture, differing payer mix and social determinants of health in the surrounding community, and even different unions. The intervention also had to be adapted to payment and market forces affecting our hospitals (e.g., unanticipated creation and expansion of observation services during the period of implementation) and vendors (e.g., vendor budget issues, including vendor bankruptcy). We believe that the need and opportunity for dynamic adaptation occurs to varying degrees in the implementation of all new programs and is not limited to the HaH implementation experience we chart in this paper [2].

We modified the protocols used in our HaH program from the existing research evidence base described in the literature. For example, the addition of new diagnoses, alteration of clinical exclusions, and the addition of 30-day postacute follow up were changes from studied protocols. Despite this, for selected findings of LOS and patient satisfaction, our outcomes were similar to those reported in the original studies in Table 1. Further, in a previous report [6], these outcomes over the period of implementation compared favorably to those of a comparison group of similar patients who were hospitalized in regular inpatient units. Thus, our implementation of an evidence-based intervention with its associated adaptations was accomplished with no obvious “program drift.”

Additionally, we made adaptations in which we modified the HaH approach to changing context each quarter. For example, we added palliative care and other programs, as well as telehealth and community paramedicine visits. However, we did not observe significant changes in effectiveness across a variety of measures. LOS, 30-day readmissions, ED visits, and patient ratings of care did not significantly change over time. Tracking and reviewing these outcomes on a monthly basis as part of a continuous improvement activity may have been important in maintaining these effects through the implementation period. In the case of escalations, we observed a trend toward higher rates in later quarters. This was expected as our clinicians purposely started the program with less complex patients and enrolled increasingly complex patients as the team gained experience. The escalation rate was actually closely monitored throughout implementation, the program team was conscious of the trend, and cases of escalation were reviewed by the team on an ongoing basis in the interest of continuous improvement. Thus, we believe that the implementation of serial adaptations may have also avoided the phenomenon of “voltage drop.”

Our study was limited by sample size by quarter. There are also occasional quarters where readmissions or other outcomes appear to increase. However, these occurrences are transient and the trend over the nine quarters is one of no change. A further limitation is that individual adaptations could be hypothesized to potentially enhance or diminish net benefits or program efficiency. For example, the community paramedicine intervention was intended to improve response time, enhance ability to adjust treatments in the home, and to reduce trips to the ED. One could hypothesize that this might enhance effectiveness. On the other hand, observation services at home were a departure from the evidence-based model, and one could hypothesize that these patients might be less likely to benefit. Because adaptations were made as the need or opportunity arose, it is not possible for us to isolate and examine the effect of any individual adaptation in this analysis.

## Conclusion

The implementation of evidence-based programs necessarily requires adaptation. We made significant adaptations to HaH at inception and then serial adaptations over the course of implementation. Over the course of the implementation, many of these important outcomes were tracked and fed back to the program leadership leading to further adaptations. Our findings may indicate that adaptations to evidence-based programs may avoid diminished benefits due to potential ‘program drift’ or ‘voltage drop’.

## Additional files

Additional file 1: Hospital at Home Baseline Survey (containing self-rated health, demographic and functional status questions). (DOCX 20 kb)

Additional file 2: Hospital at Home Two-Week Survey (containing self-rated health, readmissions, ED visit and HCAHPS questions). (DOCX 29 kb)

Additional file 3: Hospital at Home Four-Week Survey (containing self-rated health, and functional status questions). (DOCX 18 kb)

Additional file 4: Regression models controlling for patient characteristics and season of admission mentioned in page 12. (DOCX 16 kb)

## Abbreviations

ADL: Activities of Daily Living
CI: Confidence Intervals
DSF: Dynamic Sustainability Framework
ED: Emergency Department
HaH: Hospital at Home
HCAHPS: Hospital Consumer Assessment of Healthcare Providers and Systems
ICMJE: International Committee of Medical Journal Editors
IRB: Institutional Review Board
LOS: Length of Stay
OR: Odds Ratio
PPHS: Program for the Protection of Human Subjects
SE: Standard Error
