# Supplementary material for: The effect of adapting Hospital at Home to facilitate implementation and sustainment on program drift or voltage drop
Source: BMC Health Serv Res. 2019 Apr 29;19:264. doi: 10.1186/s12913-019-4063-8 (PMC6489357; doi:10.1186/s12913-019-4063-8)
Supplement: Supplementary file 1 — Hospital at Home Baseline Survey (containing self-rated health, demographic and functional status questions). (DOCX 20 kb) [file 12913_2019_4063_MOESM1_ESM.docx]

|  | MACT Evaluation  Baseline Acute |
| --- | --- |

| Q7 | Patient age:  PATIENT: How old are you?  PROXY: How old is the patient? |
| --- | --- |
|  | _______________ |

| Q8 | Patient gender: | | |
| --- | --- | --- | --- |
|  |  | ❑ | *Male* |
|  |  | ❑ | *Female* |
|  |  | ❑ | *Unknown* |

|  | General Health (SF-1) |
| --- | --- |

| Q24 | Patient's health:  PATIENT: First, I want you to think about your health one month ago. One month ago, would you say your health was...?  PROXY: First, I want you to think about *[patient name]* health one month ago. One month ago, would you say *[his/her]* health was...? | | |
| --- | --- | --- | --- |
|  |  | ❑ | *Poor* |
|  |  | ❑ | *Fair* |
|  |  | ❑ | *Good* |
|  |  | ❑ | *Very good* |
|  |  | ❑ | *Excellent* |
|  |  | ❑ | *Refused* |
|  |  | ❑ | *Don't know* |
|  |  | ❑ | *Not applicable* |

|  | Functional Status (Covinsky) |
| --- | --- |

|  | PATIENT: Now I'm going to ask you some questions about how you take care of yourself. I want you to think about your experience over the last month. Over the last month, did you do the following on your own, with some help, or were you unable to:  PROXY: Now I’m going to ask you some questions about how *[patient name]* takes care of *[himself/herself]*. I want you to think about *[his/her]* experience over the last month. Over the last month, did *[patient name]* do the following on *[his/her]* own, with some help, or was *[she/he]* unable to: |
| --- | --- |

| Q47 | Use the telephone, including looking up and dialing numbers and answering the phone? | | |
| --- | --- | --- | --- |
|  |  | ❑ | *On own/no help* |
|  |  | ❑ | *Some help* |
|  |  | ❑ | *Unable* |
|  |  | ❑ | *Refused* |
|  |  | ❑ | *Don't know* |
|  |  | ❑ | *Not applicable* |

| Q48 | PATIENT: Get to places out of walking distance by using public transportation or driving your car?  PROXY: Get to places out of walking distance by using public transportation or driving *[his/her]* car? | | |
| --- | --- | --- | --- |
|  |  | ❑ | *On own/no help* |
|  |  | ❑ | *Some help* |
|  |  | ❑ | *Unable* |
|  |  | ❑ | *Refused* |
|  |  | ❑ | *Don't know* |
|  |  | ❑ | *Not applicable* |

| Q49 | Shop for groceries or clothes? | | |
| --- | --- | --- | --- |
|  |  | ❑ | *On own/no help* |
|  |  | ❑ | *Some help* |
|  |  | ❑ | *Unable* |
|  |  | ❑ | *Refused* |
|  |  | ❑ | *Don't know* |
|  |  | ❑ | *Not applicable* |

| Q50 | PATIENT: Prepare, serve and provide meals for yourself?  PROXY: Prepare, serve and provide meals for *[himself/herself]*? | | |
| --- | --- | --- | --- |
|  |  | ❑ | *On own/no help* |
|  |  | ❑ | *Some help* |
|  |  | ❑ | *Unable* |
|  |  | ❑ | *Refused* |
|  |  | ❑ | *Don't know* |
|  |  | ❑ | *Not applicable* |

| Q51 | Do light housework, such as dusting or doing dishes? | | |
| --- | --- | --- | --- |
|  |  | ❑ | *On own/no help* |
|  |  | ❑ | *Some help* |
|  |  | ❑ | *Unable* |
|  |  | ❑ | *Refused* |
|  |  | ❑ | *Don't know* |
|  |  | ❑ | *Not applicable* |

| Q52 | Take pills or medicine in the correct amounts at the correct times? | | |
| --- | --- | --- | --- |
|  |  | ❑ | *On own/no help* |
|  |  | ❑ | *Some help* |
|  |  | ❑ | *Unable* |
|  |  | ❑ | *Refused* |
|  |  | ❑ | *Don't know* |
|  |  | ❑ | *Not applicable* |

| Q53 | PATIENT: Handle your own money, including writing checks and paying bills?  PROXY: Handle *[his/her]* own money, including writing checks and paying bills? | | |
| --- | --- | --- | --- |
|  |  | ❑ | *On own/no help* |
|  |  | ❑ | *Some help* |
|  |  | ❑ | *Unable* |
|  |  | ❑ | *Refused* |
|  |  | ❑ | *Don't know* |
|  |  | ❑ | *Not applicable* |

| Q54 | PATIENT: Washing or bathing yourself?  PROXY: Washing or bathing *[himself/herself]*? | | |
| --- | --- | --- | --- |
|  |  | ❑ | *On own/no help* |
|  |  | ❑ | *Some help* |
|  |  | ❑ | *Unable* |
|  |  | ❑ | *Refused* |
|  |  | ❑ | *Don't know* |
|  |  | ❑ | *Not applicable* |

| Q55 | Dressing or undressing? | | |
| --- | --- | --- | --- |
|  |  | ❑ | *On own/no help* |
|  |  | ❑ | *Some help* |
|  |  | ❑ | *Unable* |
|  |  | ❑ | *Refused* |
|  |  | ❑ | *Don't know* |
|  |  | ❑ | *Not applicable* |

| Q56 | Eating, including cutting food? | | |
| --- | --- | --- | --- |
|  |  | ❑ | *On own/no help* |
|  |  | ❑ | *Some help* |
|  |  | ❑ | *Unable* |
|  |  | ❑ | *Refused* |
|  |  | ❑ | *Don't know* |
|  |  | ❑ | *Not applicable* |

| Q57 | PATIENT: Walking around your home?  PROXY: Walking around *[his/her]* home? | | |
| --- | --- | --- | --- |
|  |  | ❑ | *On own/no help* |
|  |  | ❑ | *Some help* |
|  |  | ❑ | *Unable* |
|  |  | ❑ | *Refused* |
|  |  | ❑ | *Don't know* |
|  |  | ❑ | *Not applicable* |

| Q58 | PATIENT: Cleaning yourself after either bowel or bladder functions?  PROXY: Cleaning *[himself/herself]* after either bowel or bladder functions? | | |
| --- | --- | --- | --- |
|  |  | ❑ | *On own/no help* |
|  |  | ❑ | *Some help* |
|  |  | ❑ | *Unable* |
|  |  | ❑ | *Refused* |
|  |  | ❑ | *Don't know* |
|  |  | ❑ | *Not applicable* |

| Q59 | PATIENT: Over the last month, did you sometimes have an accident with your bowels or bladder either during the day or night?  PROXY: Over the last month, did *[patient name]* sometimes have an accident with *[his/her]* bowels or bladder either during the day or night? | | |
| --- | --- | --- | --- |
|  |  | ❑ | *Yes* |
|  |  | ❑ | *No* |
|  |  | ❑ | *Refused* |
|  |  | ❑ | *Don't know* |
|  |  | ❑ | *Not applicable* |

|  | Demographics (Patient) |
| --- | --- |

| Q143 | PATIENT: What is the highest grade or year of school you ever completed?  PROXY: What is the highest grade or year of school *[patient name]* ever completed? | | |
| --- | --- | --- | --- |
|  |  | ❑ | *8th grade or less* |
|  |  | ❑ | *Some high school, but did not graduate* |
|  |  | ❑ | *High school graduate or GED* |
|  |  | ❑ | *Some college or 2-year degree* |
|  |  | ❑ | *College graduate* |
|  |  | ❑ | *More than a 4-year college degree* |
|  |  | ❑ | *Refused* |

| Q144 | PATIENT: In what country were you born?  PROXY: In what country was *[patient name]* born? | | | |
| --- | --- | --- | --- | --- |
|  |  | ❑ | | *United States* |
|  |  | ❑ | | *Puerto Rico* |
|  |  | ❑ | | *Dominican Republic* |
|  |  | ❑ | | *Mexico* |
|  |  | ❑ | | *Other* |
|  |  | ❑ | | *Refused* |
|  | | | *Other (specify):* | |
|  | | | ___________________ | |

| Q145 | PATIENT: Are you Hispanic, Latino/a, or of Spanish origin?  PROXY: Is *[patient name]* Hispanic, Latino/a, or of Spanish origin? | | |
| --- | --- | --- | --- |
|  |  | ❑ | *Yes* |
|  |  | ❑ | *No* |
|  |  | ❑ | *Refused* |

| Q146 | PATIENT: Please give me the group that represents your Hispanic origin or ancestry:  PROXY: Please give me the group that represents *[patient name]*'s Hispanic origin or ancestry: | | | |
| --- | --- | --- | --- | --- |
|  |  | ❑ | | *Puerto Rican* |
|  |  | ❑ | | *Dominican* |
|  |  | ❑ | | *Mexican* |
|  |  | ❑ | | *Other* |
|  |  | ❑ | | *Refused* |
|  |  | ❑ | | *Not applicable* |
|  | | | *Specify Other:* | |
|  | | | _________________________________________ | |

| Q147 | PATIENT: How would you describe your race or ethnicity?  PROXY: How would you describe *[patient name]*'s race or ethnicity? | | | |
| --- | --- | --- | --- | --- |
|  |  | ❑ | | *White* |
|  |  | ❑ | | *Black or African American* |
|  |  | ❑ | | *American Indian or Alaskan Native* |
|  |  | ❑ | | *Asian* |
|  |  | ❑ | | *Pacific Islander* |
|  |  | ❑ | | *Other* |
|  |  | ❑ | | *Refused* |
|  |  | ❑ | | *Don't know* |
|  | | | *Specify other:* | |
|  | | | ___________________ | |

| Q148 | PATIENT: Using this card, please tell me: what is your total household monthly income, including money from a job, a pension, Social Security, SSI, or any other source? Just give me the number from the left hand column on the card.  PROXY: Using this card, please tell me: what is *[patient name]*'s total household monthly income, including money from a job, a pension, Social Security, SSI, or any other source? Just give me the number from the left hand column on the card. | | |
| --- | --- | --- | --- |
|  |  | ❑ | *(1) $500 or less per month* |
|  |  | ❑ | *(2) $501-$750 per month* |
|  |  | ❑ | *(3) $751-$1000 per month* |
|  |  | ❑ | *(4) $1001-$1350 per month* |
|  |  | ❑ | *(5) $1351-$1500 per month* |
|  |  | ❑ | *(6) $1501-$2000 per month* |
|  |  | ❑ | *(7) $2001-$2500 per month* |
|  |  | ❑ | *(8) $2501-$3000 per month* |
|  |  | ❑ | *(9) $3001 or more per month* |
|  |  | ❑ | *Refused* |
|  |  | ❑ | *Don't know* |
