# Supplementary material for: The effect of adapting Hospital at Home to facilitate implementation and sustainment on program drift or voltage drop
Source: BMC Health Serv Res. 2019 Apr 29;19:264. doi: 10.1186/s12913-019-4063-8 (PMC6489357; doi:10.1186/s12913-019-4063-8)
Supplement: Supplementary file 2 — Hospital at Home Two-Week Survey (containing self-rated health, readmissions, ED visit and HCAHPS questions). (DOCX 29 kb) [file 12913_2019_4063_MOESM2_ESM.docx]

|  | MACT Evaluation  2 Week Follow Up Acute |
| --- | --- |

|  | Intake Information |
| --- | --- |

| Q15 | PATIENT: Since your discharge from the {Q5} about two weeks ago, have you received medical care in an emergency room?  PROXY: Since *[patient name]*'s discharge from the {Q5} about two weeks ago, has *[he/she]* received medical care in an emergency room? | | |
| --- | --- | --- | --- |
|  |  | ❑ | *Yes* |
|  |  | ❑ | *No* |
|  |  | ❑ | *Don't know* |
|  |  | ❑ | *Refused* |
|  |  | ❑ | *Not applicable* |

| Q16 | PATIENT: Since your discharge from the {Q5} about two weeks ago, have you been admitted to a hospital? This does not include time you may have spent in an emergency room.  PROXY: Since *[patient name]*'s discharge from the {Q5} about two weeks ago, has *[he/she]* been admitted to a hospital? This does not include time *[he/she]* may have spent in an emergency room. | | |
| --- | --- | --- | --- |
|  |  | ❑ | *Yes* |
|  |  | ❑ | *No* |
|  |  | ❑ | *Don't know* |
|  |  | ❑ | *Refused* |
|  |  | ❑ | *Not applicable* |

|  |  |
| --- | --- |
|  | Modified HCAHPS |

|  | PATIENT: These next questions are about the care you received from nursing staff during your recent {Q5} stay.  PROXY: These next questions are about the care *[patient name]* received from nursing staff during *[his/her]* recent {Q5} stay.  PROXY and CAREGIVER: Now I have some questions about your experience during *[patient’s name]*’s {Q5} stay that began about two weeks ago. I am going to ask you about the nursing staff during *[patient’s name]*’s recent {Q5} stay.  CAREGIVER: Now I have some questions about your experience during *[patient’s name]*’s {Q5} stay that began about two weeks ago. I am going to ask you about the nursing staff during *[patient’s name]*’s recent {Q5} stay. |
| --- | --- |

| Q19 | PATIENT: During your recent {Q5} stay, how often did the nurses treat you with courtesy and respect? Would you say...  PROXY: During *[patient name]*'s recent {Q5} stay, how often did the nurses treat *[him/her]* with courtesy and respect? Would you say...  PROXY and CAREGIVER: During *[patient name]*'s recent {Q5} stay, how often did the nurses treat you with courtesy and respect? Would you say...  CAREGIVER: During *[patient name]*'s recent {Q5} stay, how often did the nurses treat you with courtesy and respect? Would you say... | | |
| --- | --- | --- | --- |
|  |  | ❑ | *Never* |
|  |  | ❑ | *Sometimes* |
|  |  | ❑ | *Usually* |
|  |  | ❑ | *Always* |
|  |  | ❑ | *Don't know* |
|  |  | ❑ | *Refused* |
|  |  | ❑ | *Not applicable* |

| Q20 | PATIENT: During your recent {Q5} stay, how often did the nurses listen carefully to you? Would you say...  PROXY: During *[patient name]*'s recent {Q5} stay, how often did the nurses listen carefully to *[him/her]*? Would you say...  PROXY and CAREGIVER: During *[patient name]*'s recent {Q5} stay, how often did the nurses listen carefully to you? Would you say...  CAREGIVER: During *[patient name]*'s recent {Q5} stay, how often did the nurses listen carefully to you? Would you say... | | |
| --- | --- | --- | --- |
|  |  | ❑ | *Never* |
|  |  | ❑ | *Sometimes* |
|  |  | ❑ | *Usually* |
|  |  | ❑ | *Always* |
|  |  | ❑ | *Don't know* |
|  |  | ❑ | *Refused* |
|  |  | ❑ | *Not applicable* |

| Q21 | PATIENT: During your recent {Q5} stay, how often did the nurses explain things in a way you could understand? Would you say...  PROXY: During *[patient name]*'s recent {Q5} stay, how often did the nurses explain things in a way *[he/she]* could understand? Would you say...  PROXY and CAREGIVER: During *[patient name]*'s recent {Q5} stay, how often did the nurses explain things in a way you could understand? Would you say...  CAREGIVER: During *[patient name]*'s recent {Q5} stay, how often did the nurses explain things in a way you could understand? Would you say... | | |
| --- | --- | --- | --- |
|  |  | ❑ | *Never* |
|  |  | ❑ | *Sometimes* |
|  |  | ❑ | *Usually* |
|  |  | ❑ | *Always* |
|  |  | ❑ | *Don't know* |
|  |  | ❑ | *Refused* |
|  |  | ❑ | *Not applicable* |

|  | *CALL BUTTON* |
| --- | --- |

| Q22 | PATIENT: Did you have an emergency or call button at your bedside during your {Q5} stay?  PROXY: Did *[patient name]* have an emergency or call button at *[his/her]* bedside during *[his/her]* {Q5} stay?  PROXY and CAREGIVER: Did *[patient name]* have an emergency or call button at *[his/her]* bedside during *[his/her]* {Q5} stay?  CAREGIVER: Did *[patient name]* have an emergency or call button at *[his/her]* bedside during *[his/her]* {Q5} stay? | | |
| --- | --- | --- | --- |
|  |  | ❑ | *Yes* |
|  |  | ❑ | *No* |
|  |  | ❑ | *Refused* |
|  |  | ❑ | *Don't know* |
|  |  | ❑ | *Not applicable* |

|  | *CALL BUTTON follow up questions* |
| --- | --- |

| Q23 | PATIENT: During your recent {Q5} stay, how often did you press the call button to contact a nurse or doctor?  PROXY: During *[patient name]*'s recent {Q5} stay, how often did *[he/she]* press the call button to contact a nurse or doctor?  PROXY and CAREGIVER: During *[patient name]*'s recent {Q5} stay, how often did you or *[patient name]* press the call button to contact a nurse or doctor?  CAREGIVER: During *[patient name]*'s recent {Q5} stay, how often did you or *[patient name]* press the call button to contact a nurse or doctor? | | |
| --- | --- | --- | --- |
|  |  | ❑ | *I never pressed the call button* |
|  |  | ❑ | *Once* |
|  |  | ❑ | *2-3 times* |
|  |  | ❑ | *4 or more times* |
|  |  | ❑ | *Don't know* |
|  |  | ❑ | *Refused* |
|  |  | ❑ | *Not applicable* |

| Q24 | PATIENT: After you pressed the call button, how often did you get help as soon as you wanted it?  PROXY: After *[patient name]* pressed the call button, how often did *[he/she]* get help as soon as *[he/she]* wanted it?  PROXY and CAREGIVER: After you or *[patient name]* pressed the call button, how often did you get help as soon as you wanted it?  CAREGIVER: After *[patient name]* pressed the call button, how often did you get help as soon as you wanted it? | | |
| --- | --- | --- | --- |
|  |  | ❑ | *Never* |
|  |  | ❑ | *Sometimes* |
|  |  | ❑ | *Usually* |
|  |  | ❑ | *Always* |
|  |  | ❑ | *Don't know* |
|  |  | ❑ | *Refused* |
|  |  | ❑ | *Not applicable* |

|  |  |
| --- | --- |

|  | PATIENT: These next questions are about the care you received from doctors during your recent {Q5} stay.  PROXY: These next questions are about the care *[patient name]* received from doctors during *[his/her]* recent {Q5} stay.  PROXY and CAREGIVER: Now I have some questions about your experience during *[patient’s name]*’s {Q5} stay that began about two weeks ago. I am going to ask you about the doctors during *[patient’s name]*’s recent {Q5} stay.  CAREGIVER: Now I have some questions about your experience during *[patient’s name]*’s {Q5} stay that began about two weeks ago. I am going to ask you about the doctors during *[patient’s name]*’s recent {Q5} stay. |
| --- | --- |

| Q25 | PATIENT: During your recent {Q5} stay, how often did the doctors treat you with courtesy and respect? Would you say...  PROXY: During *[patient name]*'s recent {Q5} stay, how often did the doctors treat *[him/her]* with courtesy and respect? Would you say...  PROXY and CAREGIVER: During *[patient name]*'s recent {Q5} stay, how often did the doctors treat you with courtesy and respect? Would you say...  CAREGIVER: During *[patient name]*'s recent {Q5} stay, how often did the doctors treat you with courtesy and respect? Would you say... | | |
| --- | --- | --- | --- |
|  |  | ❑ | *Never* |
|  |  | ❑ | *Sometimes* |
|  |  | ❑ | *Usually* |
|  |  | ❑ | *Always* |
|  |  | ❑ | *Don't know* |
|  |  | ❑ | *Refused* |
|  |  | ❑ | *Not applicable* |

| Q26 | PATIENT: During your recent {Q5} stay, how often did the doctors listen carefully to you? Would you say...  PROXY: During *[patient name]*'s recent {Q5} stay, how often did the doctors listen carefully to *[him/her]*? Would you say...  PROXY and CAREGIVER: During *[patient name]*'s recent {Q5} stay, how often did the doctors listen carefully to you? Would you say...  CAREGIVER: During *[patient name]*'s recent {Q5} stay, how often did the doctors listen carefully to you? Would you say... | | |
| --- | --- | --- | --- |
|  |  | ❑ | *Never* |
|  |  | ❑ | *Sometimes* |
|  |  | ❑ | *Usually* |
|  |  | ❑ | *Always* |
|  |  | ❑ | *Don't know* |
|  |  | ❑ | *Refused* |
|  |  | ❑ | *Not applicable* |

| Q27 | PATIENT: During your recent {Q5} stay, how often did the doctors explain things in a way you could understand? Would you say...  PROXY: During *[patient name]*'s recent {Q5} stay, how often did the doctors explain things in a way *[he/she]* could understand? Would you say...  PROXY and CAREGIVER: During *[patient name]*'s recent {Q5} stay, how often did the doctors explain things in a way you could understand? Would you say...  CAREGIVER: During *[patient name]*'s recent {Q5} stay, how often did the doctors explain things in a way you could understand? Would you say... | | |
| --- | --- | --- | --- |
|  |  | ❑ | *Never* |
|  |  | ❑ | *Sometimes* |
|  |  | ❑ | *Usually* |
|  |  | ❑ | *Always* |
|  |  | ❑ | *Don't know* |
|  |  | ❑ | *Refused* |
|  |  | ❑ | *Not applicable* |

|  |  |
| --- | --- |

|  | The next set of questions is about the {Q5} environment and your experiences. |
| --- | --- |

| Q28 | PATIENT: During your recent {Q5} stay, how often were you room and bathroom kept clean? Would you say...  PROXY and/or CAREGIVER: During *[patient name]*'s recent {Q5} stay, how often was *[his/her]* room and bathroom kept clean? Would you say... | | |
| --- | --- | --- | --- |
|  |  | ❑ | *Never* |
|  |  | ❑ | *Sometimes* |
|  |  | ❑ | *Usually* |
|  |  | ❑ | *Always* |
|  |  | ❑ | *Don't know* |
|  |  | ❑ | *Refused* |
|  |  | ❑ | *Not applicable* |

| Q29 | PATIENT: During your recent {Q5} stay, how often was the area around your room quiet at night? Would you say...  PROXY and/or CAREGIVER: During *[patient name]*'s recent {Q5} stay, how often was the area around *[his/her]* room quiet at night? Would you say... | | |
| --- | --- | --- | --- |
|  |  | ❑ | *Never* |
|  |  | ❑ | *Sometimes* |
|  |  | ❑ | *Usually* |
|  |  | ❑ | *Always* |
|  |  | ❑ | *Don't know* |
|  |  | ❑ | *Refused* |
|  |  | ❑ | *Not applicable* |

|  | *HELP GETTING TO THE BATHROOM* |
| --- | --- |

| Q30 | PATIENT: During your recent {Q5} stay, did you need help from nurses or other {Q5} staff in getting to the bathroom or in using a bedpan?  PROXY and/or CAREGIVER: During *[patient name]*'s recent {Q5} stay, did *[he/she]* need help from nurses or other {Q5} staff in getting to the bathroom or in using a bedpan? | | |
| --- | --- | --- | --- |
|  |  | ❑ | *Yes* |
|  |  | ❑ | *No* |
|  |  | ❑ | *Don't know* |
|  |  | ❑ | *Refused* |
|  |  | ❑ | *Not applicable* |

|  | *HELP GETTING TO THE BATHROOM follow up question* |
| --- | --- |

| Q31 | PATIENT: In general, how often did this help arrive in a reasonable amount of time after you asked for it?  PROXY and/or CAREGIVER: In general, how often did this help arrive in a reasonable amount of time after *[patient name]* asked for it? | | |
| --- | --- | --- | --- |
|  |  | ❑ | *Never* |
|  |  | ❑ | *Sometimes* |
|  |  | ❑ | *Usually* |
|  |  | ❑ | *Always* |
|  |  | ❑ | *Don't know* |
|  |  | ❑ | *Refused* |
|  |  | ❑ | *Not applicable* |

|  | *PAIN MANAGEMENT* |
| --- | --- |

| Q32 | PATIENT: During your recent {Q5} stay, did you need medicine for pain?  PROXY and/or CAREGIVER: During *[patient name]*'s recent {Q5} stay, did *[he/she]* need medicine for pain? | | |
| --- | --- | --- | --- |
|  |  | ❑ | *Yes* |
|  |  | ❑ | *No* |
|  |  | ❑ | *Don't know* |
|  |  | ❑ | *Refused* |
|  |  | ❑ | *Not applicable* |

|  | *PAIN MANAGEMENT follow up questions* |
| --- | --- |

| Q33 | PATIENT: During your recent {Q5} stay, how often was your pain well controlled? Would you say...  PROXY and/or CAREGIVER: During *[patient name]*'s recent {Q5} stay, how often was *[his/her]* pain well controlled? Would you say... | | |
| --- | --- | --- | --- |
|  |  | ❑ | *Never* |
|  |  | ❑ | *Sometimes* |
|  |  | ❑ | *Usually* |
|  |  | ❑ | *Always* |
|  |  | ❑ | *Don't know* |
|  |  | ❑ | *Refused* |
|  |  | ❑ | *Not applicable* |

| Q34 | PATIENT: During your recent {Q5} stay, how often did the {Q5} staff do everything they could to help you with your pain? Would you say...  PROXY and/or CAREGIVER: During *[patient name]*'s recent {Q5} stay, how often did the {Q5} staff do everything they could to help *[him/her]* with your pain? Would you say... | | |
| --- | --- | --- | --- |
|  |  | ❑ | *Never* |
|  |  | ❑ | *Sometimes* |
|  |  | ❑ | *Usually* |
|  |  | ❑ | *Always* |
|  |  | ❑ | *Don't know* |
|  |  | ❑ | *Refused* |
|  |  | ❑ | *Not applicable* |

|  | *NEW MEDICATIONS* |
| --- | --- |

| Q35 | PATIENT: During your recent {Q5} stay, were you given any medicine that you had not taken before?  PROXY and/or CAREGIVER: During *[patient name]*'s recent {Q5} stay, was *[he/she]* given any medicine that *[he/she]* had not taken before? | | |
| --- | --- | --- | --- |
|  |  | ❑ | *Yes* |
|  |  | ❑ | *No* |
|  |  | ❑ | *Don't know* |
|  |  | ❑ | *Refused* |
|  |  | ❑ | *Not applicable* |

|  | *NEW MEDICATIONS follow up questions* |
| --- | --- |

| Q36 | PATIENT: Before giving you any new medicine, how often did {Q5} staff tell you what the medicine was for? Would you say...  PROXY: Before giving *[patient name]* any new medicine, how often did {Q5} staff tell *[him/her]* what the medicine was for? Would you say...  PROXY and CAREGIVER: Before giving *[patient name]* any new medicine, how often did {Q5} staff tell you what the medicine was for? Would you say...  CAREGIVER: Before giving *[patient name]* any new medicine, how often did {Q5} staff tell you what the medicine was for? Would you say... | | |
| --- | --- | --- | --- |
|  |  | ❑ | *Never* |
|  |  | ❑ | *Sometimes* |
|  |  | ❑ | *Usually* |
|  |  | ❑ | *Always* |
|  |  | ❑ | *Don't know* |
|  |  | ❑ | *Refused* |
|  |  | ❑ | *Not applicable* |

| Q37 | PATIENT: Before giving you any new medicine, how often did {Q5} staff describe possible side effects in a way you could understand? Would you say...  PROXY: Before giving *[patient name]* any new medicine, how often did {Q5} staff describe possible side effects in a way *[he/she]* could understand? Would you say...  PROXY and CAREGIVER: Before giving *[patient name]* any new medicine, how often did {Q5} staff describe possible side effects in a way you could understand? Would you say...  CAREGIVER: Before giving *[patient name]* any new medicine, how often did {Q5} staff describe possible side effects in a way you could understand? Would you say... | | |
| --- | --- | --- | --- |
|  |  | ❑ | *Never* |
|  |  | ❑ | *Sometimes* |
|  |  | ❑ | *Usually* |
|  |  | ❑ | *Always* |
|  |  | ❑ | *Don't know* |
|  |  | ❑ | *Refused* |
|  |  | ❑ | *Not applicable* |

|  |  |
| --- | --- |

|  | The next set of questions are about when you were discharged from the {Q5}. |
| --- | --- |

| Q38 | PATIENT: After you were discharged from the {Q5}, did you stay in your own home, return to your own home, go to someone else's home, or go to another health facility?  PROXY and/or CAREGIVER: After *[patient name]* was discharged from the {Q5}, did *[he/she]* stay in *[his/her]* own home, return to *[his/her]* own home, go to someone else's home, or go to another health facility? | | |
| --- | --- | --- | --- |
|  |  | ❑ | *Stayed in own home / Returned to own home* |
|  |  | ❑ | *[blank] - do not select* |
|  |  | ❑ | *Someone else's home* |
|  |  | ❑ | *Another health facility* |
|  |  | ❑ | *Don't know* |
|  |  | ❑ | *Refused* |
|  |  | ❑ | *Not applicable* |

| Q39 | PATIENT: During your recent {Q5} stay, did doctors, nurses, or other {Q5} staff talk with you about whether you would have the help you needed when you left {Q5}?  PROXY: During *[patient name]*'s recent {Q5} stay, did doctors, nurses, or other {Q5} staff talk with *[patient name]* about whether *[he/she]* would have the help *[he/she]* needed when *[he/she]* left {Q5}?  PROXY and CAREGIVER: During *[patient name]*'s recent {Q5} stay, did doctors, nurses, or other {Q5} staff talk with you about whether you would have the help you needed when *[he/she]* left {Q5}?  CAREGIVER: During *[patient name]*'s recent {Q5} stay, did doctors, nurses, or other {Q5} staff talk with you about whether you would have the help you needed when *[he/she]* left {Q5}? | | |
| --- | --- | --- | --- |
|  |  | ❑ | *Yes* |
|  |  | ❑ | *No* |
|  |  | ❑ | *Don't know* |
|  |  | ❑ | *Refused* |
|  |  | ❑ | *Not applicable* |

| Q40 | PATIENT: During your recent {Q5} stay, did you get information in writing about what symptoms or health problems to look out for after you left {Q5}?  PROXY: During *[patient name]*'s recent {Q5} stay, did *[he/she]* get information in writing about what symptoms or health problems to look out for after *[he/she]* left {Q5}?  PROXY and CAREGIVER: During *[patient name]*'s recent {Q5} stay, did you get information in writing about what symptoms or health problems to look out for after *[he/she]* left {Q5}?  CAREGIVER: During *[patient name]*'s recent {Q5} stay, did you get information in writing about what symptoms or health problems to look out for after *[he/she]* left {Q5}? | | |
| --- | --- | --- | --- |
|  |  | ❑ | *Yes* |
|  |  | ❑ | *No* |
|  |  | ❑ | *Don't know* |
|  |  | ❑ | *Refused* |
|  |  | ❑ | *Not applicable* |

|  | This next question is about the {Q5} stay that ended about two weeks ago. Please do not include any other hospital stays in your answer. |
| --- | --- |

| Q41 | PATIENT: We want to know your overall rating of your experience in the {Q5}. On a scale of 0 to 10, what would you rate the {Q5}...?  PROXY: We want to know *[patient name]*'s overall rating of your experience in the {Q5}. On a scale of 0 to 10, what would *[patient name]* rate the {Q5}...?  PROXY and CAREGIVER: We want to know your overall rating of your experience with the {Q5}. On a scale of 0 to 10, what would you rate the {Q5}...?  CAREGIVER: We want to know your overall rating of your experience with the {Q5}. On a scale of 0 to 10, what would you rate the {Q5}...? | | | | | | | | | | | | | | | | | | | | | | | | | | | |
| --- | --- | --- | --- | --- | --- | --- | --- | --- | --- | --- | --- | --- | --- | --- | --- | --- | --- | --- | --- | --- | --- | --- | --- | --- | --- | --- | --- | --- |
|  |  | *0* |  | *1* |  | *2* |  | *3* |  | *4* |  | *5* |  | *6* |  | *7* |  | *8* |  | *9* |  | *10* |  | *DK* |  | *Ref* |  | *NA* |
|  | Where 0 is the worst possible hospital experience and 10 is the best possible hospital experience | ❑ |  | ❑ |  | ❑ |  | ❑ |  | ❑ |  | ❑ |  | ❑ |  | ❑ |  | ❑ |  | ❑ |  | ❑ |  | ❑ |  | ❑ |  | ❑ |

| Q42 | PATIENT: Would you recommend the {Q5} to your friends and family if they needed it? Would you say...  PROXY: Would *[patient name]* recommend the {Q5} to *[his/her]* friends and family if they needed it? Would you say...  PROXY and CAREGIVER: Would you recommend the {Q5} to your friends and family if they needed it? Would you say...    CAREGIVER: Would you recommend the {Q5} to your friends and family if they needed it? Would you say... | | |
| --- | --- | --- | --- |
|  |  | ❑ | *Definitely no* |
|  |  | ❑ | *Probably no* |
|  |  | ❑ | *Probably yes* |
|  |  | ❑ | *Definitely yes* |
|  |  | ❑ | *Don't know* |
|  |  | ❑ | *Refused* |
|  |  | ❑ | *Not applicable* |

|  | General Health (SF-1) |
| --- | --- |

| Q46 | Patient's health:  PATIENT: First, I want you to think about your health one month ago. One month ago, would you say your health was...?  PROXY: First, I want you to think about *[patient name]* health one month ago. One month ago, would you say *[his/her]* health was...? | | |
| --- | --- | --- | --- |
|  |  | ❑ | *Poor* |
|  |  | ❑ | *Fair* |
|  |  | ❑ | *Good* |
|  |  | ❑ | *Very good* |
|  |  | ❑ | *Excellent* |
|  |  | ❑ | *Refused* |
|  |  | ❑ | *Don't know* |
|  |  | ❑ | *Not applicable* |
