# Supplementary material for: The effect of adapting Hospital at Home to facilitate implementation and sustainment on program drift or voltage drop
Source: BMC Health Serv Res. 2019 Apr 29;19:264. doi: 10.1186/s12913-019-4063-8 (PMC6489357; doi:10.1186/s12913-019-4063-8)
Supplement: Supplementary file 3 — Hospital at Home Four-Week Survey (containing self-rated health, and functional status questions). (DOCX 18 kb) [file 12913_2019_4063_MOESM3_ESM.docx]

|  | MACT Evaluation  4 Week Acute |
| --- | --- |

|  |  |
| --- | --- |
|  |  |

|  | General Health (SF-1) |
| --- | --- |

| Q46 | Patient's health:  PATIENT: First, I want you to think about your health one month ago. One month ago, would you say your health was...?  PROXY: First, I want you to think about ***[patient name]*** health one month ago. One month ago, would you say ***[his/her]*** health was...? | | |
| --- | --- | --- | --- |
|  |  | ❑ | *Poor* |
|  |  | ❑ | *Fair* |
|  |  | ❑ | *Good* |
|  |  | ❑ | *Very good* |
|  |  | ❑ | *Excellent* |
|  |  | ❑ | *Refused* |
|  |  | ❑ | *Don't know* |
|  |  | ❑ | *Not applicable* |

|  | Functional Status (Covinsky) |
| --- | --- |

|  | PATIENT: Now I'm going to ask you some questions about how you take care of yourself. I want you to think about your experience over the last month. Over the last month, did you do the following on your own, with some help, or were you unable to:  PROXY: Now I’m going to ask you some questions about how ***[patient name]*** takes care of ***[himself/herself]***. I want you to think about ***[his/her]*** experience over the last month. Over the last month, did ***[patient name]*** do the following on ***[his/her]*** own, with some help, or was ***[she/he]*** unable to: |
| --- | --- |

| Q57 | Use the telephone, including looking up and dialing numbers and answering the phone? | | |
| --- | --- | --- | --- |
|  |  | ❑ | *On own/no help* |
|  |  | ❑ | *Some help* |
|  |  | ❑ | *Unable* |
|  |  | ❑ | *Refused* |
|  |  | ❑ | *Don't know* |
|  |  | ❑ | *Not applicable* |

| Q58 | PATIENT: Get to places out of walking distance by using public transportation or driving your car?  PROXY: Get to places out of walking distance by using public transportation or driving ***[his/her]*** car? | | |
| --- | --- | --- | --- |
|  |  | ❑ | *On own/no help* |
|  |  | ❑ | *Some help* |
|  |  | ❑ | *Unable* |
|  |  | ❑ | *Refused* |
|  |  | ❑ | *Don't know* |
|  |  | ❑ | *Not applicable* |

| Q59 | Shop for groceries or clothes? | | |
| --- | --- | --- | --- |
|  |  | ❑ | *On own/no help* |
|  |  | ❑ | *Some help* |
|  |  | ❑ | *Unable* |
|  |  | ❑ | *Refused* |
|  |  | ❑ | *Don't know* |
|  |  | ❑ | *Not applicable* |

| Q60 | PATIENT: Prepare, serve and provide meals for yourself?  PROXY: Prepare, serve and provide meals for ***[himself/herself]***? | | |
| --- | --- | --- | --- |
|  |  | ❑ | *On own/no help* |
|  |  | ❑ | *Some help* |
|  |  | ❑ | *Unable* |
|  |  | ❑ | *Refused* |
|  |  | ❑ | *Don't know* |
|  |  | ❑ | *Not applicable* |

| Q61 | Do light housework, such as dusting or doing dishes? | | |
| --- | --- | --- | --- |
|  |  | ❑ | *On own/no help* |
|  |  | ❑ | *Some help* |
|  |  | ❑ | *Unable* |
|  |  | ❑ | *Refused* |
|  |  | ❑ | *Don't know* |
|  |  | ❑ | *Not applicable* |

| Q62 | Take pills or medicine in the correct amounts at the correct times? | | |
| --- | --- | --- | --- |
|  |  | ❑ | *On own/no help* |
|  |  | ❑ | *Some help* |
|  |  | ❑ | *Unable* |
|  |  | ❑ | *Refused* |
|  |  | ❑ | *Don't know* |
|  |  | ❑ | *Not applicable* |

| Q63 | PATIENT: Handle your own money, including writing checks and paying bills?  PROXY: Handle ***[his/her]*** own money, including writing checks and paying bills? | | |
| --- | --- | --- | --- |
|  |  | ❑ | *On own/no help* |
|  |  | ❑ | *Some help* |
|  |  | ❑ | *Unable* |
|  |  | ❑ | *Refused* |
|  |  | ❑ | *Don't know* |
|  |  | ❑ | *Not applicable* |

| Q64 | PATIENT: Washing or bathing yourself?  PROXY: Washing or bathing ***[himself/herself]***? | | |
| --- | --- | --- | --- |
|  |  | ❑ | *On own/no help* |
|  |  | ❑ | *Some help* |
|  |  | ❑ | *Unable* |
|  |  | ❑ | *Refused* |
|  |  | ❑ | *Don't know* |
|  |  | ❑ | *Not applicable* |

| Q65 | Dressing or undressing? | | |
| --- | --- | --- | --- |
|  |  | ❑ | *On own/no help* |
|  |  | ❑ | *Some help* |
|  |  | ❑ | *Unable* |
|  |  | ❑ | *Refused* |
|  |  | ❑ | *Don't know* |
|  |  | ❑ | *Not applicable* |

| Q66 | Eating, including cutting food? | | |
| --- | --- | --- | --- |
|  |  | ❑ | *On own/no help* |
|  |  | ❑ | *Some help* |
|  |  | ❑ | *Unable* |
|  |  | ❑ | *Refused* |
|  |  | ❑ | *Don't know* |
|  |  | ❑ | *Not applicable* |

| Q67 | PATIENT: Walking around your home?  PROXY: Walking around ***[his/her]*** home? | | |
| --- | --- | --- | --- |
|  |  | ❑ | *On own/no help* |
|  |  | ❑ | *Some help* |
|  |  | ❑ | *Unable* |
|  |  | ❑ | *Refused* |
|  |  | ❑ | *Don't know* |
|  |  | ❑ | *Not applicable* |

| Q68 | PATIENT: Cleaning yourself after either bowel or bladder functions?  PROXY: Cleaning ***[himself/herself]*** after either bowel or bladder functions? | | |
| --- | --- | --- | --- |
|  |  | ❑ | *On own/no help* |
|  |  | ❑ | *Some help* |
|  |  | ❑ | *Unable* |
|  |  | ❑ | *Refused* |
|  |  | ❑ | *Don't know* |
|  |  | ❑ | *Not applicable* |

| Q69 | PATIENT: Over the last month, did you sometimes have an accident with your bowels or bladder either during the day or night?  PROXY: Over the last month, did ***[patient name]*** sometimes have an accident with ***[his/her]*** bowels or bladder either during the day or night? | | |
| --- | --- | --- | --- |
|  |  | ❑ | *Yes* |
|  |  | ❑ | *No* |
|  |  | ❑ | *Refused* |
|  |  | ❑ | *Don't know* |
|  |  | ❑ | *Not applicable* |

| Q14 | PATIENT: Since we last spoke 2 weeks ago, have you had an unscheduled or urgent visit in your home or a doctor's office to receive medical care? This does not include a visit that was scheduled for you at the time of discharge.  PROXY: Since we last spoke 2 weeks ago, has***[he/she]*** had an unscheduled or urgent visit in your home or a doctor's office to receive medical care? This does not include a visit that was scheduled for ***[him/her]*** at the time of discharge. | | |
| --- | --- | --- | --- |
|  |  | ❑ | *Yes* |
|  |  | ❑ | *No* |
|  |  | ❑ | *Don't know* |
|  |  | ❑ | *Refused* |
|  |  | ❑ | *Not applicable* |

| Q15 | PATIENT: Since we last spoke 2 weeks ago, have you received medical care in an emergency room?  PROXY: Since we last spoke 2 weeks ago, has***[he/she]*** received medical care in an emergency room? | | |
| --- | --- | --- | --- |
|  |  | ❑ | *Yes* |
|  |  | ❑ | *No* |
|  |  | ❑ | *Don't know* |
|  |  | ❑ | *Refused* |
|  |  | ❑ | *Not applicable* |

| Q16 | PATIENT: Since we last spoke 2 weeks ago, have you been admitted to a hospital? This does not include time you may have spent in an emergency room.  PROXY: Since we last spoke 2 weeks ago, has***[he/she]*** been admitted to a hospital? This does not include time ***[he/she]*** may have spent in an emergency room. | | |
| --- | --- | --- | --- |
|  |  | ❑ | *Yes* |
|  |  | ❑ | *No* |
|  |  | ❑ | *Don't know* |
|  |  | ❑ | *Refused* |
|  |  | ❑ | *Not applicable* |
