# Supplementary material for: The effect of adapting Hospital at Home to facilitate implementation and sustainment on program drift or voltage drop
Source: BMC Health Serv Res. 2019 Apr 29;19:264. doi: 10.1186/s12913-019-4063-8 (PMC6489357; doi:10.1186/s12913-019-4063-8)
Supplement: Supplementary file 4 — Regression models controlling for patient characteristics and season of admission mentioned in page 12. (DOCX 16 kb) [file 12913_2019_4063_MOESM4_ESM.docx]

Addition file 4

|  | Length  of Stay  *parameter estimate (se)* | Escalation  *odds ratio (95% CI)* | 30 day Hospital Readmission  *odds ratio (95% CI)* | 30 day  ED visit  *odds ratio (95% CI)* | Overall Rating  of care  *odds ratio (95% CI)* |
| --- | --- | --- | --- | --- | --- |
| Age | -0.00 (0.00) | 0.99 (0.96, 1.01) | 1.01 (0.99,1.08) | 1.03 (1.01, 1.05)** | 0.98 (0.97,0.99)** |
| Male | -0.53 (0.12)** | 0.66 (0.41, 1.05) | 1.22 (0.81,1.84) | 1.48 (0.88, 2.47) | 0.52 (0.37,0.74)** |
| Race/ Ethnicity |  |  |  |  |  |
| African American | -0.62 (0.16)** | 1.31 (0.78, 2.19) | 2.04 (1.19,3.48)** | 2.01 (1.00, 4.01)* | 1.39 (0.87,2.22) |
| Hispanic | 0.14 (0.17) | 0.86 (0.48, 1.53) | 1.79 (0.97,3.30) | 1.06 (0.54, 2.11) | 1.87 (1.14,3.07)* |
| Education |  |  |  |  |  |
| Some high school | -0.48 (0.16)** | 1.31 (0.73,2.31) | 3.52 (1.71, 7.27)** | 3.82 (1.50, 9.75)** | 1.31 (0.83,2.08) |
| High school graduate | -0.55 (0.19)** | 1.06 (0.53,2.10) | 2.32 (1.00, 5.35)* | 8.59 (3.30, 22.40)** | 0.84 (0.50,1.41) |
| Some college | -0.19 (0.20)** | 0.95 (0.45,1.99) | 7.79 (3.23,18.28)** | 4.01 (1.33, 12.08)* | 0.57 (0.31,1.02) |
| Period Admitted |  |  |  |  |  |
| Spring | 0.09 (0.16) | 0.79 (0.46, 1.36) | 1.40 (0.83,2.39) | 1.26 (0.53, 2.96) | 0.81 (0.53, 1.24) |
| Summer | 0.18 (0.15) | 0.95 (0.58, 1.53) | 0.99 (0.58,1.17) | 4.19 (2.00, 8.77)** | 1.69 (1.09,2.65)* |
| Autumn | 0.21 (0.18) | 1.03 (0.56,1.90) | 0.91 (0.46,1.79) | 1.31 (0.51, 3.33) | 0.88 (0.55,1.43) |
| Insurance |  |  |  |  |  |
| Private | -0.48 (0.18)** | 0.53 (0.25,1.13) | 1.53 (0.82,2.82) | 6.33 (3.30, 12.15)** | 0.65 (0.38,1.11) |
| Medicaid | 0.15 (0.15) | 1.27 (0.77,2.12) | 1.59 (0.93,2.74) | 2.47 (1.29, 4.73)** | 0.51 (0.32,0.80)** |
| Activities of Daily Living | 0.19 (0.13) | 0.63 (0.41,0.98)* | 1.27 (0.79,2.05) | 1.07 (0.59, 1.94) | 1.23 (0.86,1.80) |
| Self-Reported General Health |  |  |  |  |  |
| Poor | 0.26 (0.11)* | 0.59 (0.41,0.88)** | 1.11 (0.75,1.65) | 1.65 (0.99, 2.76) | 1.65 (1.20,2.27)** |
| Quarter | 0.00 (0.02) | 1.09 (1.01,1.18)* | 1.00 (0.93,1.08) | 0.93 (0.86, 1.01) | 0.99 (0.93,1.05) |

*p <0.05, **p<0.01
